# Improving the role of global conservation treaties in addressing contemporary threats to lions

**DOI:** 10.1007/s10531-018-1567-1

**Published:** 2018-06-02

**Authors:** Timothy Hodgetts, Melissa Lewis, Hans Bauer, Dawn Burnham, Amy Dickman, Ewan Macdonald, David Macdonald, Arie Trouwborst

**Affiliations:** 1 Wildlife Conservation Research Unit (Panthera et al.), University of Oxford, Tubney, UK; 2 Department of European and International Public Law, Tilburg University, Tilburg, The Netherlands

**Keywords:** International law, Treaties, *Panthera leo*, Threats, Implementation, Reform

## Abstract

Despite their iconic status, lion (*Panthera leo*) populations continue to decline across the majority of their range. In the light of the recent decision (in October 2017) to add lions to the Appendices of the Convention on Migratory Species (CMS), this paper identifies the new and existing legal protections afforded to lions through five global treaties, and maps these protections against the most critical contemporary threats facing the species. It thus offers a new analysis of the CMS listing, and draws on existing legal reviews, to highlight the ways in which global treaties offer differing forms of protection for lions. It then combines multiple concordant assessments of lion populations, to highlight nine categories of threat: human-lion conflict, bushmeat poaching, human encroachment, trophy hunting, trade in lion bones, unpredictable environmental events, socio-economic factors, policy failures, and governance/institutional weakness. The paper assesses how the various treaties each address these different categories of threat. The analysis identifies two pathways for improving legal protection: expanding the application of global treaties in respect of lions and their habitats (the paper considers the CMS listing in these terms), and improving the implementation of treaty commitments through local and national-scale actions. Furthermore, it identifies local implementation challenges that include the local knowledge of rules, compliance with rules and enforcement capacity, alongside the variety in local contexts and situations, and suggests where global treaties might provide support in meeting these challenges. We suggest that this analysis has wider implications for how treaty protection can and is utilised to protect various species of large-bodied, wide-ranging animals.

## Introduction

After a ground-breaking Conference of the Parties (COP) in Manila in October 2017, the lion (*Panthera leo*) (among 34 species, including leopards (*Panthera pardus*)) was added to the Appendices of the Convention on Migratory Species of Wild Animals (CMS), even though 4 range states opposed the listing, and several of these indicated their intention to enter reservations. This addition signals the need for states to develop a more elaborate and coordinated species-specific framework for lion conservation—and a first step towards achieving this was made through Parties’ decision to establish an African Carnivores Initiative in collaboration with the Convention on International Trade in Endangered Species of Wild Fauna and Flora (CITES). In this paper, we consider how the CMS listing of lions (on Appendix II) combines with the existing international legal architecture for lion conservation, to address the various threats that the species faces—and, importantly, where this architecture falls short, given the specific nature of those threats. We suggest that our analysis has wider relevance for mammalian wildlife (especially large carnivores), through identifying the various roles played by global treaties—and their omissions—in addressing the varied threats that endanger large animals.

International treaties provide important frameworks for wildlife conservation worldwide (Bowman et al. 2010; Trouwborst et al. 2017a), and markedly so for charismatic carnivores (Trouwborst 2015). For large carnivores their wide-ranging ecology, reliance on large prey species, and intersection with global trade networks often requires cross-border cooperation for effective conservation action (Ripple et al. 2014). Moreover, large carnivore species tend to attract significant attention from publics and politicians (Lorimer 2015; Macdonald et al. 2016) and have the potential to raise awareness for a wide range of other threatened species (Macdonald et al. 2017b). Yet collectively these species are in trouble despite their ecological and cultural importance (Ripple et al. 2014), and lions (Bauer et al. 2015) are no exception. Measuring population sizes accurately is notoriously difficult, but recent estimates for lions suggest that numbers have rapidly declined, with between 20,000 and 30,000 lions remaining across Africa (Bauer et al. 2016). The conservation of lions also has an added dimension insofar as they are a geographically widely distributed species (albeit occupying a fraction of their historical range), and measures directed towards their conservation may result in various benefits to other species (Macdonald et al. 2012). Therefore, legal instruments designed to conserve lions may thus also cast a protective shadow over wider biodiversity.

In this transdisciplinary paper we (a team of legal experts, conservation biologists and social scientists) draw on the findings of two reviews concerning lions and international law (Trouwborst et al. 2017c; Watts 2016), combined with our analysis of recent developments under the CMS, to map global wildlife treaties against the various ecological and socio-political threats this species faces. We demonstrate that while the coverage of such treaties across lion range states is reassuring, the use of these treaties to address the current threats faced by lions is patchy at best—even before we consider the daunting practicalities of implementation, monitoring and enforcement. Of course, international treaties are only one lever within wider conservation strategies, and will not always be the most appropriate instruments for addressing specific threats. Furthermore, in this paper we limit our scope to the global treaties, and do not assess the roles played by regional agreements and protocols in various African contexts. Nevertheless, we suggest improvements are possible and worthwhile pursuing. We note two pathways to improve the contribution of international law to lion conservation: (i) adapting and expanding the application of existing treaties, recent examples of which are the listing of, and associated decisions regarding, lions by the CMS COP (Conference of the Parties) in 2017, and (ii) improving the local-level implementation of treaties.

## The relevance of international treaties for lions

**Fig. 1:**
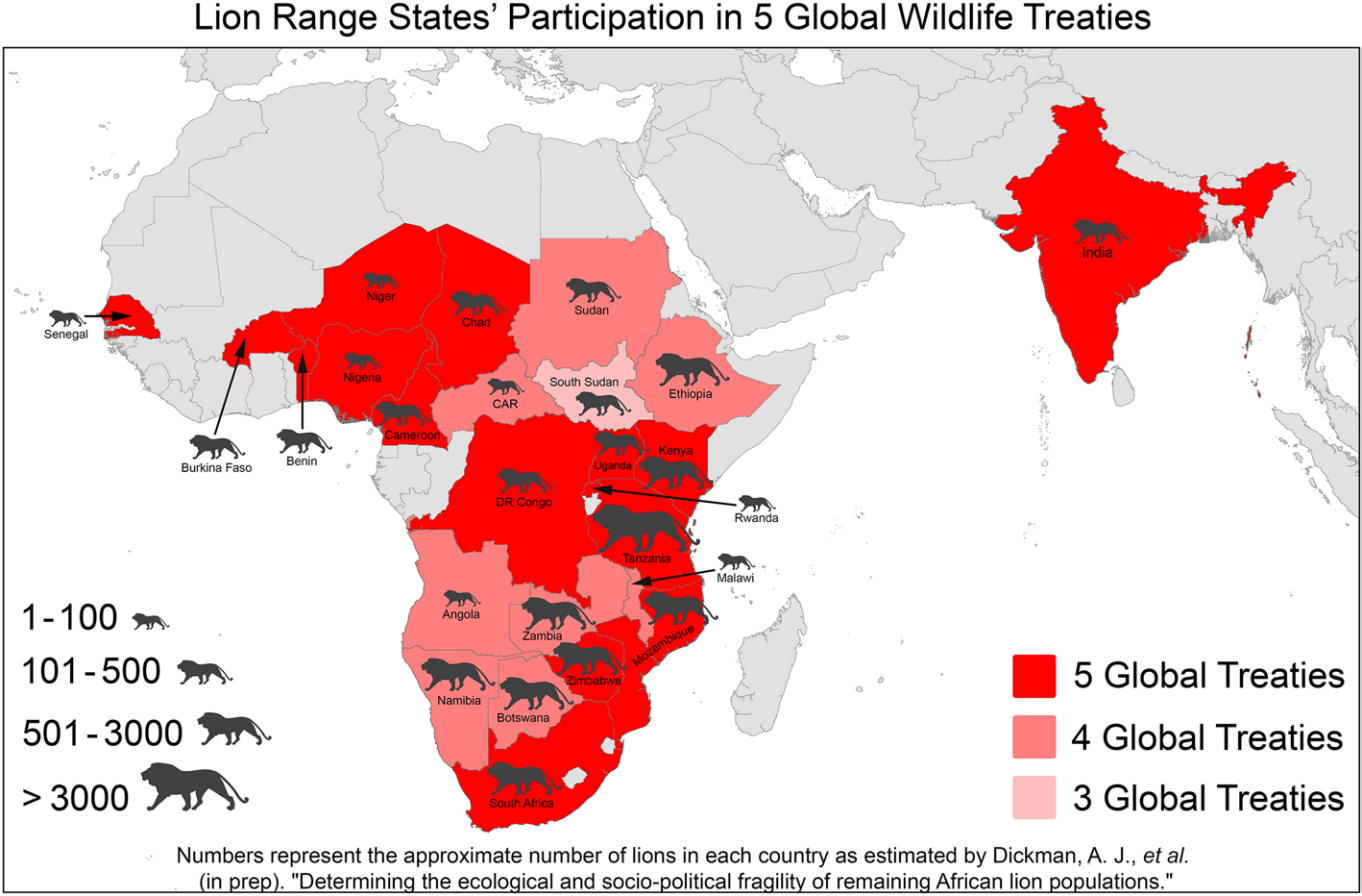
Lion range states’ participation in 5 global wildlife treaties. (Color figure online)

All contemporary lion range states are contracting parties to three or more of the ‘Big 5’ global conservation treaties that—amongst other things—obligate, support, regulate and coordinate measures beneficial to lions and their prey and habitat, either directly or indirectly (Fig. 1). These are: the Convention on Biological Diversity, the ‘Ramsar’ Convention on Wetlands of International Importance, the World Heritage Convention, CITES, and the CMS.

Below, we summarize the relevance of each global treaty for lions. There are also several relevant treaties with a more limited geographic scope, but they are not the subject of this analysis. These include the 1968 African Convention on the Conservation of Nature and Natural Resources (and the 2003 revision thereof); sub-regional instruments like the SADC Protocol on Wildlife Conservation and Law Enforcement; treaty-based regional entities like the Commission of Central African Forests (COMIFAC), the East African Community (EAC) and the Economic Community of West African States (ECOWAS); and treaties establishing transfrontier conservation areas across Africa. Some of the most relevant of these agreements are discussed in (Trouwborst et al. 2017c), which considered 33 lion range states, including countries where lions have either been present in recent history, or where there is speculation that they may continue to persist. Here we limit our analysis to known contemporary populations, as listed in the IUCN Red List (25 African states plus India).

### Convention on Biological Diversity (CBD), 1992

The CBD is a broad-ranging treaty. It is concerned with the conservation and sustainable use of biodiversity, and amongst other things includes general obligations to create protected areas, support threatened species, and create biodiversity strategies and action plans. It has played an important role in gaining additional political support for furthering habitat protection, globally. However, the text of the CBD is, from a legal perspective, so broadly worded as to make the thresholds for compliance somewhat ambiguous (Trouwborst 2015). The CBD also provides a forum for capacity building, discussion, and norm-creation around global biodiversity issues. The resulting high level biodiversity strategies, and guidance documents on more specific issues, have clear relevance for lion conservation. For example, the CBD’s Aichi Biodiversity Targets set clear goals around conserving threatened species, and the Convention has played a leading role in developing recommendations on addressing the unsustainable taking of bushmeat. All contemporary lion range states are parties.

### Ramsar Wetlands Convention, 1971

The longstanding global wetlands convention provides a basis for lion conservation through requiring that parties formulate and implement their planning so as to promote the conservation of a specified list of wetland habitats of international importance (‘Ramsar sites’) designated by individual contracting parties, and as far as possible promote the ‘wise use’ of all wetlands. Certain Ramsar sites are important habitats for lions, and for 24 sites (across 13 countries) their importance for lions (amongst other species) is part of the documentation justifying designation. Ramsar designation thus confers a layer of internationally recognised protection for certain key wetland areas in addition to any other protective designation the site may have (as a reserve, national park etc.), and the potential benefits of support provided under the Convention. All contemporary lion range states, except Angola and Ethiopia, are parties.

### World Heritage Convention (WHC), 1972

Similarly to Ramsar, some important lion habitat is protected through the WHC. Examples of large, iconic sites include the Serengeti National Park in Tanzania, and the Okavango Delta in Botswana. In total, 15 sites in the 25 contemporary lion range states cover over 1,30,000 km^2^ of lion range (Trouwborst et al. 2017c). In contrast to the Ramsar Convention, the list of sites designated under the WHC is determined centrally by the World Heritage Committee (constituting a selection of parties on rotation), following nominations by the states in which these sites occur. Furthermore, the condition of World Heritage Sites is monitored: sites can be deemed ‘in danger’ (relevant to 4 of the 15 at present), and ultimately WHC status can be removed. Given the international prestige (and tourism) driven by WHC status, the threat of removal creates an enforcement mechanism that the Ramsar Convention largely lacks. Furthermore, the WHC, which is embedded within UNESCO, can provide technical support and guidance to improve the management of ‘at risk’ sites. All contemporary lion range states are parties.

### Convention on International Trade in Endangered Species (CITES), 1973

CITES is relevant to lion conservation with respect to trade in lion body parts and trophy hunting. The treaty aims to prevent the over-exploitation of species, and works through regulating international trade in live animals, body parts and derivative products. Species that are threatened or at risk of becoming threatened and are (potentially) affected by international trade are listed in the Convention’s Appendices, with Appendix I species (species threatened with extinction which are, or may be, affected by trade) being subject to greater restrictions than those on Appendix II (species which might become threatened with extinction if trade is not regulated). Captive-bred animals from Appendix I species are treated as Appendix II—which has been an important consideration in recent discussions around lions. At present, African lions are listed on Appendix II, with the Indian population on Appendix I. There have been two recent proposals from some lion range states to ‘uplist’ all lions to Appendix I, by Kenya in 2004 and by nine states from West and Central Africa ahead of the 2016 CITES COP. However, this is an area of contention amongst lion range states, particularly due to the possible impact of uplisting on trophy hunting, although it is possible for some trophy hunting to continue under an Appendix I listing. Notably, CITES’ ban on the international trade of Appendix I species applies only to specimens which, once imported, would be used for “primarily commercial purposes”, and the Convention additionally allows exemptions from its ordinary provisions for specimens which constitute “personal or household effects”. Under certain complex, and strictly limited, conditions it is therefore possible for some international movement of hunting trophies to continue under an Appendix I listing (as an example, see CITES Res. Conf. 10.14 (Rev. CoP16) on hunting trophies for leopards).

While the COP negotiations did not ultimately result in a new Appendix I status for all lions, the meeting did agree on a new ‘annotation’ to the Appendix II listing for African lions which effectively prohibited international trade in most types of lion body parts from wild populations, although allowing regulated, quota-controlled trade for body parts deriving from captive-bred lions. Annotations are used to define the scope of a species’ inclusion on one of the CITES Appendices and can (as was the case for the lion) result in an Appendix II species being subject to more stringent trade restrictions than are ordinarily attached to this Appendix.

The COP also adopted resolutions about trophy hunting (suggesting sustainable and well-managed activities can contribute to species conservation) and demand reduction strategies to address illegal wildlife trade, as well as several decisions on the African lion concerning the broader lion conservation agenda (addressing joint conservation planning, capacity building, research and education, among other things). All contemporary lion range states are currently parties to CITES, with the exception of South Sudan, which in any event participated as a non-party in the most recent CITES COP. The Convention has developed various mechanisms to facilitate and, if need be, compel compliance—including through the imposition of trade sanctions.

### Convention on Migratory Species of Wild Animals (Bonn Convention, CMS), 1979

The CMS, which is administered by the United Nations Environment Programme (UNEP), applies predominantly to ‘migratory species’, which are defined as those species where a ‘significant proportion’ of their members ‘cyclically and predictably cross one or more national jurisdictional boundaries’. The CMS COP has taken a remarkably flexible approach to interpreting this term, accepting that ‘cyclically’ ‘relates to a cycle of any nature, such as astronomical (circadian, annual etc.), life or climatic, and of any frequency’, and that ‘predictably’ ‘implies that a phenomenon can be anticipated to recur in a given set of circumstances, though not necessarily regularly in time’ (Res. 11.33). Depending on their conservation status, species which satisfy this definition can be listed on either (or both) of two appendices. The Convention identifies concrete conservation measures that parties must take in respect of the endangered migratory species listed in Appendix I—with ‘endangered’ being interpreted as meaning ‘facing a very high risk of extinction in the wild in the near future’, and as primarily encompassing species with an EW, CE or EN status on the IUCN Red List (Res. 11.33). Controversially, the measures required in respect of Appendix I-listed species include prohibiting the deliberate killing of these species, subject to limited exceptions. Such exceptions must be precise as to content and limited in space and time, should ‘not operate to the disadvantage of the species’, and may be made only (a) for scientific purposes, (b) for purposes of ‘enhancing the propagation or survival’ of the species, (c) to ‘accommodate the needs of traditional subsistence users’ of the species, or (d) if ‘extraordinary circumstances so require’, (CMS, Article III(5)). The Convention also provides a framework for developing tailor-made mechanisms to support the conservation of specific species/groups of species—including the migratory species listed on Appendix II (the emphasis of this appendix being on migratory species with an unfavourable conservation status which require international cooperation for their conservation and management), as well as any species whose members periodically cross international borders. Such mechanisms may take a variety of forms, ranging from legally binding ancillary treaties to non-binding Memoranda of Understanding, ‘Concerted Actions’, International Species Action Plans and ‘Special Species Initiatives’. Although their use is not restricted to listed species, listing arguably increases the likelihood that a species will receive attention under the Convention by raising its profile and providing a clear mandate to the CMS Secretariat to dedicate time and resources to supporting its conservation.

In 2014, the 11th CMS COP recognized that lions are ‘migratory’ for the purposes of the Convention (Res. 11.32); and at the following meeting of the COP, in Manila in October 2017, a decision was made to add lions to Appendix II of the treaty. Basing their arguments on home range utilization, dispersal, movements following herbivore migrations, and movements as a result of annual climatic conditions, the proponents of the listing (Chad, Niger and Togo) asserted that the lion’s transboundary movements are sufficiently cyclical and predictable for the purposes of listing. They further identified countries sharing lion populations suspected to cyclically and predictably traverse national boundaries, such that a significant portion of lions could be considered ‘migratory’ for CMS purposes (UNEP/CMS/COP12/Doc.25.1.3 2017). Despite earlier consensus (Res. 11.32), this argument became controversial, and four parties (South Africa, Tanzania, Uganda and Zimbabwe) opposed the listing, arguing, *inter alia*, that transborder movements do not necessarily equate to migration and that the lion’s movements fail to satisfy all elements of the Convention’s definition of ‘migratory species’. Ultimately, the matter had to be decided by vote, this being the first vote on a species’ listing in the history of the Convention. The listing was adopted by a large majority of the parties present and voting, with only the aforementioned four states voting against. Notably, the text of Resolution 11.33 was reconsidered at the 2017 COP, but its interpretation of ‘migratory species’ remained unchanged. To the extent that arguments exist for modifying the Convention’s flexible approach to listing, it would (for the sake of consistency) appear to be more appropriate to raise these in the context of a general discussion on whether to revise this Resolution than in discussions over whether or not to list individual species that happen to be of political interest. To date, however, such a discussion has not emerged. The majority of listing proposals have been adopted without contention (indeed, there are instances in which species considerably less migratory than lions have been listed without debate), with the agreed interpretation of ‘migratory species’ tending only to be questioned when high-profile listings are at stake. In the absence of a broader discussion of Resolution 11.33, each listing of a species that is transboundary but does not migrate in the classical sense adds weight to future arguments that the Convention’s appendices are not reserved exclusively for species that engage in predictable annual migrations. The lion’s listing is especially significant in this regard insofar as the majority of CMS parties were willing to explicitly endorse a flexible approach, even when confronted with fierce arguments against it.

Although the CMS allows for listing to be applied selectively at the level of geographically separate populations, it is significant that the proponents of the lion’s Appendix II listing were not prepared to exclude the lion populations of South Africa, Tanzania, Uganda and Zimbabwe, which are not biologically distinct from contiguous populations. Indeed, such an exclusion of parts of populations using ecologically arbitrary boundaries—international frontiers between countries—would have been contrary to the spirit of the Convention and would have set a bad precedent for future listing decisions. It is further notable that the CMS definition of ‘migratory species’ only applies to wild animals, and thus has no application to captive populations. Several lion populations are intensively managed and some were reintroduced after local extirpation (e.g. Akagera in Rwanda); South Africa distinguishes ‘wild’, ‘wild managed’ and ‘captive’ populations (Bauer et al. 2016; Funston and Levendal 2014; Miller et al. 2013). Although CMS listing would exclude captive populations, all other populations would putatively be included—a precedent for this being established when Przewalski’s horse (*Equus ferus przewalskii*) was listed without any reference to it having been extinct in the wild for several decades and then reintroduced, using captive individuals, and managed (UNEP/CMS/COP12/Doc.25.1.8).

In addition to listing the lion on Appendix II, the 2017 CMS COP adopted several decisions on this species (which corresponded to the decisions adopted at the most recent CITES COP) and agreed on the establishment of a joint CMS-CITES African Carnivores Initiative to support the implementation of resolutions and decisions on lions, leopards, cheetahs (*Acinonyx jubatus*) and wild dogs (*Lycaon pictus*) under both treaties (CMS 2017). Theoretically, the Initiative could ultimately pave the way for the development of a more formal arrangement, such as a carnivore-focused treaty. However, this is unlikely in the foreseeable future, given the Convention’s recent shift away from the costly development of new legal and administrative frameworks in an attempt to channel more resources directly into implementation support. Notably, range states which are not parties to the CMS can still choose to participate in ancillary instruments, action plans and initiatives. Two existing multi-species CMS Concerted Actions are likely to become relevant to lions (Central Eurasian Aridland Mammals, Sahelo-Saharan Megafauna) and a third has been mooted as a possibility (Sub-Saharan Megafauna). The lion population in India could also potentially be included in the Convention’s Central Asian Mammals Initiative. The majority of lion range states are parties to the CMS, exceptions are: Botswana, Central African Republic, Malawi, Namibia, South Sudan, Sudan and Zambia.

### Assessing cumulative coverage of global treaties

Global treaties thus support the conservation of contemporary populations of lions in various ways. Furthermore, the majority of lion range states are parties to the treaties—all for the CBD and the WHC, all except South Sudan for CITES and all except Angola and Ethiopia for the Ramsar Convention. Even the CMS, which has the lowest level of coverage, still has 17 of 25 lion range states as parties (accounting for 80% of the estimated contemporary lion species population).

## Mapping the contributions of global conservation treaties against contemporary threats

The various forms of legal protection conferred through global treaties apply to the majority of lion range states and extant lion populations. However, they presently do not comprehensively and directly address the most critical threats facing lions in Africa (99% of the worldwide population). We rely on three recent sources to collate threats to lions: (i) the Communiqué from the May 2016 CMS-CITES hosted meeting of African lion range states in Entebbe, (ii) a recent report on Africa’s lions published by Panthera, WildAid and the WildCRU, Oxford (Panthera et al. 2016), and (iii) the IUCN’s Red List assessments (Bauer et al. 2016; Breitenmoser et al. 2008). Our synthesis of these three sources is in close accord with other recent assessments (Lindsey et al. 2017). The full list of threats is summarized in Table 1, with the relevance of each treaty noted for each threat. Whilst these threats vary in their severity for different regions and sub-populations, the summary herein is for lions in the aggregate.

**Table 1:** Summary of how global treaties address threats to lions

Threats	Severity of threat	Treaty	Relevance
Human-lion conflict (retaliatory and pre-emptive killing) [i,ii,iii]	Critical	CBD	Provides general requirement and platform to address threat
		RAMSAR	Requires and facilitates addressing threat for certain wetlands
		WHC	Requires and facilitates addressing threat for certain sites
		CMS	Provides species-specific platform for coordination and targeted action
		CITES	Provides species-specific platform for coordination and targeted action
Bushmeat poaching (prey depletion) [i,ii,iii]	Critical	CITES	Requires and facilitates regulation of international bushmeat trade
		CBD	Provides general requirement and platform to address threat
		RAMSAR	Requires and facilitates addressing threat for certain wetlands
		WHC	Requires and facilitates addressing threat for certain sites
		CMS	Provides species-specific platform for coordination and targeted action
Human encroachment/habitat degradation [i,ii,iii]	High	CBD	Provides general requirement and platform to address threat
		RAMSAR	Requires and facilitates addressing threat for certain wetlands
		WHC	Requires and facilitates addressing threat for certain sites
		CMS	Provides species-specific platform for coordination and targeted action
Poorly regulated trophy hunting [ii,iii]	Medium	CITES	Regulates international movements of trophies
		CBD	Provides general requirement and platform to ensure sustainability
		RAMSAR	Requires ensuring sustainability of hunting for certain wetlands
		WHC	Requires ensuring sustainability of hunting for certain sites
		CMS	Provides species-specific platform for coordination and targeted action
Trade in lion bones [i,ii,iii]	Unknown	CITES	Regulates international trade (currently prohibited, except captive-bred)
		CBD	Provides general requirement to promote sustainability of hunting
		RAMSAR	Requires avoiding harmful impact for certain wetlands
		WHC	Requires avoiding harmful impact for certain sites
		CMS	Provides species-specific platform for coordination and targeted action
Unpredictable events (e.g., floods) [iv]	Not quantified	CBD	Provides general requirement to promote resilience
		RAMSAR	Requires and facilitates ensuring resilience for certain wetlands
		WHC	Requires and facilitates ensuring resilience for certain sites
		CMS	Provides species-specific platform for coordination and targeted action
Socio-economic factors [i]	Not quantified	CITES	Requires and facilitates ensuring sustainability of trade
		CBD	Requires and facilitates sustainable use
		RAMSAR	Requires and facilitates wise use of wetlands
		WHC	Requires and facilitates sustainable use of natural heritage
		CMS	Provides species-specific platform for coordination and targeted action
Policy failure and political factors [i]	Not quantified	CITES	Provides platform for coordination regarding international trade
		CBD	Provides general platform for coordination
		RAMSAR	Provides platform for coordination regarding wetlands
		WHC	Provides platform for coordination regarding natural heritage
		CMS	Provides species-specific platform for coordination and targeted action
Governance and institutional weakness [i]	Not quantified	CITES	Promotes capacity-building regarding international trade
		CBD	Promotes capacity-building regarding biodiversity generally
		RAMSAR	Promotes capacity-building regarding wetlands
		WHC	Promotes capacity-building regarding natural heritage
		CMS	Provides species-specific platform for capacity-building

Sources for threat analysis given below. ‘Severity of threat’ from Panthera et al. (2016)

(i) The Entebbe Communiqué from the May 2016 CMS-CITES hosted meeting of African Range States. (ii) Panthera et al. (2016), (iii) Bauer et al. IUCN (2016), (iv) Breitenmoser et al. IUCN (2008)

The most pressing issues facing African lions are human-lion conflict leading to retaliatory or pre-emptive killing, and bushmeat poaching leading to prey depletion: these threats are ranked as critical in the Panthera-led report, followed by human encroachment which is ranked as a high threat (Panthera et al. 2016). At present, these issues are addressed by global treaties only to a modest extent. The CBD provides a general requirement and platform to address these threats. In addition, the Ramsar and World Heritage Conventions require and facilitate addressing these threats for certain designated sites. Both the CBD and CITES COPs have also adopted resolutions on the issue of bushmeat hunting, and the 2017 CMS COP adopted several decisions to address the unsustainable use of bushmeat (‘wild meat’, in CMS parlance). While the various convention texts do not address human-wildlife conflict specifically, a considerable body of guidance has been developed on the issue by the various COPs, in documents that are not themselves legally binding but can nevertheless influence the interpretation and application of the binding treaty provisions (also known as ‘soft law’). More importantly, they also address some of the causes (e.g., regulating livestock access to protected areas) and some symptoms (e.g., the indiscriminate killing of lions). However, given the urgency of the threats, and the failure to address them at present, it is worth considering the additional roles global treaties might play. As discussed above, the recent listing of lions in Appendix II of the CMS has created a legal framework through which a variety of actions might be taken that could focus specifically on these issues, and could coordinate action across the remaining range states. We note the significant potential of the CMS-CITES African Carnivores Initiative in this regard, with a substantial part of the Initiative’s envisaged budget earmarked for promoting human-carnivore coexistence (UNEP/CMS/COP12/Doc.24.3.1.1). That such frameworks might be accessible to non-party range states (see earlier discussion) adds significantly to their potential.

The other threats to lions are addressed more directly by global treaties. Habitats gain a degree of legal protection from human encroachment and resulting degradation through the international designation of certain areas (with accompanying conservation benefits) under the auspices of the WHC and Ramsar, and the broadly-phrased CBD commitment to establish protected areas and to protect endangered species and habitats. However, the continuing severity of the threats to lion habitat (Riggio et al. 2013) raises important questions as to the scope of such ‘generalist’ (i.e., not species-specific) treaties, and the procedures and mechanisms by which enforcement of treaty provisions might occur (Trouwborst et al. 2017c). Both the international movement of hunting trophies and trade in lion bones are addressed directly by CITES, although important debates and discussions continue as to whether the current arrangements (summarized earlier) are effective (see discussion). Stochastic events such as floods and fires can pose a threat to lions in small, geographically restricted populations, and remain a significant threat for the population of lions in India (Breitenmoser et al. 2008); again, the indirect support for protected areas provided by treaties is relevant here, as is the CMS objective of maintaining unfragmented, more robust and resilient transboundary populations. The Entebbe Communiqué also recognized a series of socio-political threats relating to governance, policy, and economic issues that combine to limit the efficacy of lion conservation. The various global treaties are particularly important when it comes to policy formulation and capacity building in this respect. The bureaucracies of the various treaties, grounded *inter alia* in UNEP and UNESCO, hold significant potential as regards institutional support and coordination for lion conservation.

As many species besides lions are covered by the various global wildlife treaties, some of which face similar threats to lions, several aspects of the preceding analysis concerning these treaties will also apply to their actual or potential roles with regard to such species, particularly other large carnivores (Trouwborst 2015).

## Discussion

Becoming a party to international treaties is an important way that lion range states can signal their commitment to manage their wildlife populations sustainably, and the publicity around these treaties raises much-needed global awareness of the plight of lions and other species. However, despite the widespread applicability of global treaties across lion range, lions have continued to decline calamitously: across Africa, their numbers have approximately halved in the past 20 years, and they have been extirpated from over 90% of their historic range (Bauer et al. 2016). Clearly, therefore, there is a pressing need to improve the performance of international treaties in terms of effective lion conservation. Here, we suggest two main ways that lion conservation could be improved under the global treaties discussed in this paper:

(i) Adapt and expand the application of existing treaties
(ii) Improve local-level implementation of treaties

### Adapt and expand the application of existing treaties

First, existing treaties might be better utilised for lion conservation (Trouwborst 2015). The recent listing of lions in Appendix II of the CMS is an example. This listing raises the lion’s international legal profile whilst simultaneously providing bureaucratic capacity and a framework to support additional coordinated action by lion range states to address the most critical threats to the species. However, international agreement by itself will not be enough to combat prey depletion, and policing the killing of large carnivores is resource intensive and difficult (Critchlow et al. 2016; Johnson et al. 2016). Thus, a package of measures is needed that improves both the legal framework *and* local implementation and enforcement (see below).

Another reform might involve CITES. There is an important debate occurring as to whether the current treatment of lions in CITES is optimal (Bauer et al. 2018; Trouwborst et al. 2017c). Future actions planned by CITES, resulting from the 2016 COP17, include the creation of a Task Force on African Lions and the formulation and implementation of conservation strategies for the species, in collaboration with the CMS. Yet significant questions continue to revolve around whether allowing legal markets for megafauna body parts from captive populations is compatible with reducing demand for illegally-sourced body parts (Di Minin et al. 2015), whether controlling a chain of custody for legal products is practically feasible (Bennett 2015) and whether look-alike issues create insurmountable enforcement problems across species. The extent of trade in body parts of wild lions has been a cause of concern, and although initial expert surveys do not reveal it as a serious problem at present, it may become so in the future (Williams et al. 2015a, b, 2017a, b). There is also continuing debate around the status of trophy hunting within lion conservation, and whether it should be considered as a threat to wild populations or as a sustainable form of management and source of conservation finance (Di Minin et al. 2016; Macdonald et al. 2016, 2017a).

Further ways to increase the utility of global wildlife treaties include the listing of additional sites of importance to lion conservation under the Ramsar and World Heritage Conventions (Trouwborst et al. 2017c). It would also be beneficial to more fully utilise the support mechanisms associated with these Conventions to improve the management of currently designated sites, so that they can better conserve lion populations. Funding has been identified as a major issue in lion conservation (Lindsey et al. 2017; Packer et al. 2013); global treaties can mobilise funding but can also provide guidance and bureaucratic capacity in spending and administering it. This is not an easy task as, in practice, ‘improving management’ involves complex social and ecological challenges. As an example, the Ngorongoro Conservation Area (NCA) is a World Heritage Site, and has received over US$270 000 in international assistance since its designation in 1979 (UNESCO 2015). The NCA has experienced intense human-lion conflict (associated with a rapidly growing human population) and the lion population has declined substantially since the 1980s. There are mounting tensions regarding the management of the NCA, a multiple land-use conservation area where humans (mainly Maasai pastoralists), lions and other wildlife are intended to coexist. UNESCO and the IUCN conducted a reactive monitoring mission there in 2008, noting that while a previous study stated that the area had a carrying capacity of 25 000 people with cattle, 67,000 people lived in the NCA by 2007 (UNESCO 2015). However, the Maasai were not involved in the carrying capacity study and contested the findings. Moreover, many of the Maasai in the area have given up their nomadic lifestyles and are now dependent upon agriculture, but all forms of agriculture were banned in the NCA in 2007 (Ikanda and Packer 2008). The ban led to much antagonism amongst local residents, although it has been neither enforced nor managed. UNESCO’s World Heritage Centre and the IUCN consider that enforcing the ban would be positive for conservation of the site, but it would clearly affect the livelihoods of local people, especially as even the 25,000 estimate of carrying capacity involved some reliance upon agriculture. Conflicts between local Maasai communities and the NCA authorities are growing, particularly as residents feel disempowered. The World Heritage Centre and IUCN have made several recommendations for action, including commissioning a new carrying capacity study explicitly considering the needs of the local Maasai community, encouraging the voluntary relocation of immigrants, and increasing the active participation of local communities in the decision-making processes. Thus, international treaties and their bureaucratic apparatuses can play a role in improving local management, but doing so involves navigating these complex social and ecological considerations.

### Improve local-level implementation of international treaties

The value of international treaties for conservation is multifaceted (Trouwborst et al. 2017a), but their impact remains limited where they are poorly implemented, for example due to corruption or other poor governance practices. The provisions of international treaties are often transposed into national legal systems through domestic laws and regulations. Treaties thus impose obligations on states, but it is up to those states to utilise or create national arrangements to meet their obligations. Many of the treaty obligations are broad rather than very specific, and the party involved is expected to do what works best, which includes striking the right balance between top-down legislation and bottom-up approaches (Redpath et al. 2017). Since the fates of lions are largely determined by the actions of resident or nomadic people living alongside them, sensitively designed conservation strategies at both a national and sub-national level are vital to ensuring that the obligations of international treaties are met, while ensuring that the rules are applicable and appropriate for the local people concerned. Conversely, implementation of treaties can be ineffective in a variety of ways, notably: if compliance with, awareness of or enforcement of national laws and regulations is lacking; if bottom-up conservation strategies fail to meet parties’ obligations; and/or if parties’ national implementation laws and other conservation actions are themselves inadequate for complying with international obligations.

For domestic measures to be effective in meeting treaty obligations, it is important that people understand the different legal categories for wildlife, and that there are effective management plans and communication strategies regarding the rules associated with those categories. In many lion range countries, some or all of those components are lacking. All too often local communities are entirely unacquainted with national laws or regulations protecting wildlife, as they are often disengaged from the legal and conservation discourse. For example, in Tanzania’s Ruaha landscape, which still supports the world’s second largest lion population but has extremely high rates of lion killing (Abade et al. 2014), local people are usually completely unaware that lions are in decline, and are also largely unaware of domestic laws designed to protect them (Dickman pers. obs.). In Mozambique, a country with 15% of remaining lion range, a study in Maputo Elephant Reserve found that 65% of people (82% of whom were dependent upon consumptive wildlife use) had never heard of the country’s new Forest and Wildlife Policy (Soto et al. 2001). Moving outside of lion range states, a study in Madagascar revealed that less than half the local respondents were able to correctly classify species into their legal categories (Keane et al. 2011). This study further suggested that engaging people directly in natural resource management can sensitise them to conservation laws, which is likely to have relevance in many lion areas, where those people living alongside lions are often marginalised and report very little engagement in any conservation activities (Dickman 2009). Furthermore, increasing local awareness of *international* requirements can result in enhanced support for conservation measures. For instance, in a survey of the benefits associated with the designation of Ramsar sites in Africa, for some sites international designation had led to increased interest by local communities, the establishment of community patrols to reduce trespassing and poaching, and/or in a change of behaviour of site users (Gardner et al. 2008).

Even when knowledge of locally applicable rules and regulations is apparent, these are frequently ignored. For example, Tanzania, which has 40% of the world’s remaining lions and over a quarter of remaining lion range, has severe human-lion conflict and very high rates of anthropogenic lion killing, with just one study documenting 35 lions killed around 3 small villages in less than 18 months (Dickman 2015). Lion killings in Tanzania often fulfil both a ritual role, to prove bravery, and a defensive one to reduce attacks on livestock or people (Dickman 2015; Ikanda and Packer 2008; Kissui 2008). While Tanzania banned ritual lion hunting in the 1970s, the country’s 2009 Wildlife Conservation Act (amended in 2013) permits people to kill lions and other wildlife in defence of human life or livestock; if people are aware of legal restrictions they are also aware of this loophole. Lions impose major costs on local people (Dickman 2009; Packer et al. 2005), so additional measures to reduce conflict are necessary. Much lion killing is centred around livestock losses, which are particularly devastating for traditional pastoralist societies, where cattle have high cultural as well as economic value. Unless international treaty obligations can be translated into more effective livestock protection, such threats will continue. However, lion killing often has multiple drivers, such as the cultural status that people get from killing lions, or the fact it can offer one of the few ways of generating wealth (usually through gifts of cattle from herders in thanks for removing a predator) or facilitating marriages, and this can generate sufficient social and economic benefits for lion killing to persist even in the near-absence of livestock attacks (Fitzherbert et al. 2014). Understanding these dynamics is fundamentally important to developing effective conservation strategies, and requires the involvement of local people to ascertain site-specific threats and determine which legal measures would be most effective at combating them. Engaging communities in decision-making and ownership of control mechanisms has led to proven success in some lion areas: in Namibia, enabling local people to own and control their wildlife has contributed to one of the few increasing national lion populations, while in areas of Kenya, Tanzania, Ethiopia and Mozambique, locally-driven programmes where communities develop and enforce rules have proven relatively successful at reducing threats to lions (Fitzherbert et al. 2014; Gebresenbet et al. 2017; Hazzah et al. 2014; Redpath et al. 2017). It is important to note, therefore, that the involvement of local people, participatory approaches, poverty alleviation, awareness-raising and education have become increasingly central tenets of all the major conservation treaties, as reflected in COP decisions, strategies, allocation of funding, manuals and other technical guidance. Useful examples of the latter include the Ramsar Handbook on Participatory Skills (Ramsar Convention Secretariat 2010), the World Heritage Resource Manual on Managing Natural World Heritage (UNESCO et al. 2012), and the CBD Technical Series volume on livelihood alternatives for unsustainable bushmeat use (Secretariat of the Convention on Biological Diversity 2011).

The efficacy of national measures taken to implement international commitments is also determined by the ability of range states to monitor and enforce the ensuing regulations, and on the quality of the laws and regulations themselves. Laws may be flouted due to a low probability of detecting illegal behaviour, particularly in countries which have limited capacity within their wildlife agencies (Shanee 2012). Many wildlife crimes fail to be detected by the authorities; data from Zambia’s Luangwa Valley suggested that detection rates of black rhinoceros (*Diceros bicornis*) and elephant (*Loxodonta africana*) poaching were too low to prevent declines (Leader-Williams and Milner-Gulland 1993), while a study across four countries by Akella and Cannon (2004) estimated that the risk of punishment for environmental crime was very low (below 1% in the two countries with reasonable estimates). This is supported by our experience in lion range countries: in Ruaha, over 90% of the documented lion killing events were detected by researchers, with authorities having very little capacity to monitor anthropogenic killing outside (and sometimes even within) protected areas (Dickman pers. obs.). Wildlife rangers often have little training, and commonly resort to lethal control as a first option for resolving human-lion conflict. Such methods tend to reinforce local attitudes assuming killing lions is the best way to manage conflict, and thus frequently lead to additional subsequent lion killing (Dickman pers. obs.). In addition to compliance and enforcement, the parties’ national implementation laws must be fit for purpose in the first place. This is not always the case. For example, the central bureaucracy of CITES monitors the extent to which contracting parties’ national legislation (and related measures) are able to implement the treaty’s provisions, and this differs markedly between lion range states (CITES 2016). National-level implementation capacity is affected by a range of socio-political, economic and cultural factors that shape the efficacy of national and sub-national bureaucratic activities—what might be termed a nation’s ‘conservation likelihood’(Dickman et al. 2015).

As a final example of implementation challenges, the proliferation of ‘paper parks’ which are not effectively funded, managed or enforced is well documented (Bruner et al. 2001). Furthermore, the efficacy of protected areas can be additionally compromised by changes in governance arrangements, including to the human activities allowed therein, the size of the area, or even the removal of protected status—trends termed ‘PADDD’ or protected area downgrading, downsizing and degazettement in the literature (Mascia and Pailler 2011), and of potential relevance in some lion range states (see www.padddtracker.org for details). Many lion range states have insufficient funds to effectively manage core protected areas (Lindsey et al. 2017), so providing sufficient law enforcement resources and expanding effective wildlife conservation on human-dominated land is unlikely without significant additional investment. International treaties have the capacity to leverage additional funding for protected areas, as well as wider wildlife conservation, so could play a very valuable role in this regard. Yet even with the stricter monitoring and enforcement provisions of the WHC noted earlier, many natural World Heritage Sites are threatened by the ecological isolation caused by development outside their borders (Allan et al. 2017). Such edge effects, including those relating to the pressures of human populations, are especially relevant for lions given the threats (such as bushmeat hunting and human-lion conflict) they currently face (Woodroffe and Ginsberg 1998). Furthermore, action beyond their boundaries is often necessary even if protected areas are well managed, and even though lions are increasingly limited to protected areas some range lies outside of reserves; Durant et al. (2017) actually demonstrated that survival of some species depends on non-protected land, including the cheetah and African wild dog that have most of their range outside reserves. Addressing local concerns and community practices is a complex undertaking that requires a multi-faceted conservation strategy (Redpath et al. 2017); but as discussed herein, global treaties may help when it comes to building capacity and developing guidance in these respects.

### Concluding observations

When it comes to international law, like all forms of wildlife law, the details of interpretation matter (Epstein et al. 2016; Trouwborst et al. 2017b). So do the contextual specificities of the range states to which treaty provisions apply: their politics, governance, bureaucratic capacity, histories, cultures, and socio-economic characteristics (Dickman et al. 2015). The review of global treaties offered here is a high-level summary, and as such the intricacies of how each treaty has been and might be further utilised (or not) to address specific threats to lions in different regions and cultural contexts are not addressed in great detail (see Trouwborst et al. 2017c). Furthermore, legal measures, whether created through international treaties, national laws, local or community restrictions, are only ever a part of a broader set of measures within conservation. However, even limiting the review to this high level, we have suggested several priorities for reform and practical pathways towards improving lion conservation. These are: expanding the application of global treaties in respect of lions and their habitats (with the recent CMS listing representing a potentially significant step in these terms), improving the local-level implementation of treaty commitments through participatory regulatory developments, and supporting implementation and enforcement capacity at national and local scales. In conclusion, even if their effective implementation is beset with practical challenges, global wildlife treaties have distinct contributions to make to lion conservation, the importance of which is likely to increase rather than diminish in the foreseeable future.
